# Effectiveness of the four-component protein-based meningococcal vaccine against *Neisseria gonorrhoeae* infections: Mounting evidence and public health implications for Canada

**DOI:** 10.14745/ccdr.v51i08a04

**Published:** 2025-08-28

**Authors:** Philippe De Wals, Yen-Giang Bui, Michaël Desjardins

**Affiliations:** 1 Département de médecine sociale et préventive, Université Laval, Québec City, QC; 2 Institut national de santé publique du Québec, Québec City, QC; 3 Division des maladies infectieuses, Centre hospitalier de l’Université de Montréal, Montréal, QC; 4 Département de microbiologie, infectiologie et immunologie, Faculté de médecine, Université de Montréal, Montréal, QC

**Keywords:** meningococcal vaccine, gonorrhea, immunization program, high-risk groups

## Abstract

**Background:**

In Canada, the burden of gonorrhea has been increasing steadily over the last decade with emerging multi-drug-resistant strains. There is a high genomic similarity between *Neisseria meningitidis* and *Neisseria gonorrhoea*.

**Methods:**

Review of published studies and on-going trials with the four-component meningococcal serogroup B vaccine (4CMenB–Bexsero®).

**Results:**

Observational studies have shown protection against gonorrhea infection ranging from 35% to 59% for up to three years after the administration of 4CMenB. Several randomized clinical trials are also under way. Results from the DOXYVAC trial have been published but the sample size was too small to exclude a protective effect in the 30%–50% range. Recommendations on the use of 4CMenB for individuals at high risk of gonorrhea infection have been issued in the United Kingdom and New York state based on results of observational studies.

**Conclusion:**

If results of observational studies are confirmed by randomized trials with an acceptable cost-effectiveness profile in the Canadian context, a targeted immunization program using 4CMenB could be implemented.

## Introduction

In Canada, the burden of gonorrhea has been increasing steadily over the last decade, with a reported rate almost tripling from 2010 (33.5 per 100,000 population) to 2019 (94.3 per 100,000 population) (1,2). Because of the COVID-19 pandemic, the years 2020 and 2021 were atypical, with reduced travel and contacts among individuals, hesitancy to seek medical care, and diagnostic test shortages ((3)). Males account for 56% of all cases diagnosed and the most commonly affected age group consists of those 15 to 39 years old, accounting for 82% of total cases ((2)). The incidence of sexually transmitted infections is particularly high in street youth, sex workers, men who have sex with men, users of hard drugs, incarcerated persons, and some Indigenous people (4). The proportion of multi-drug-resistant strains has also increased from 8.6% in 2015 to 12.4% in 2019, a source of concern for treatment effectiveness ((2)).

In this commentary, results of studies on the impact of the four-component meningococcal serogroup B (4CMenB) vaccination on gonorrhea risk are presented, along with the biological plausibility and cost-effectiveness analyses. The public health implications of these findings are discussed in terms of product information and possible recommendations for specific high-risk groups in Canada.

## Methods

A review was performed of published observational studies and on-going trials on the protein-based 4CMenB (Bexsero®, Glaxo-Smith-Kline) aiming to protect against *Neisseria gonorrhoeae* infection and disease. A PubMed search was performed on October 5, 2023, using the following combination of terms: (meningococcal vaccine OR 4CMenB) AND gonorrhea. A total of 121 hits was obtained. Titles and abstracts were reviewed using inclusion and exclusion criteria. Inclusion criteria were quantitative studies in humans aiming to evaluate the effect of vaccination on the occurrence of *N. gonorrhoeae* infection by comparing 4CMenB vaccination with a control group. An exclusion criterion was any ecological study design using before-after comparisons without ascertainment of the immunization status of each individual. When several manuscripts described results of a same study, the latest analysis was selected. A similar search was performed in Google Scholar, PubMed and Clinicaltrial.gov and results were completed by information provided by the 4CMenB manufacturer.

## Results

### Epidemiological studies

In 2009, a mass immunization campaign was implemented in the Saguenay–Lac-Saint-Jean region of Québec to control an increase of serogroup B invasive meningococcal disease using 4CMenB. The vaccination campaign reached 86% of the target population aged six months to 20 years, with most receiving two doses ((5)). Following the mass campaign, an unexpected decrease in the number of gonococcal infections among persons aged ≤20 years was observed in the region ((6)). Such a decrease was not seen among adults aged >20 years, and there was also a clear continuing upward trend in the number of *Chlamydia trachomatis* infections in all age groups. The review identified five other observational studies on this issue and results described in **Table 1** support the hypothesis of a cross-protection against gonorrhea generated by 4CMenB in the 35% to 59% range for up to three years after vaccination (7–11).

**Table 1:** Observational studies aiming to assess the effectiveness of the four-valent serogroup B meningococcal vaccine (4CMenB) against gonorrhea

Reference	Setting	Study design	Main results
Wang *et al.,* 2023 ((7))	In 2018, a publicly funded 4CMenB program was introduced in South Australia: infants are offered three doses, and two doses for grade 10 school students (about 15 years of age).	Vaccine impact was assessed using a Poisson or negative binomial regression model, and vaccine effectiveness (VE) was estimated using screening and case-control methods. Chlamydia controls were used to control potential confounding effects such as high-risk sexual behaviour associated with sexually transmitted infections.	Two-dose VE was 33.2% (95% CI: 15.9%–47.0%). The VE estimate after 36 months post-vaccination was 23.2% (95% CI: 0%–47.5%) compared to 34.9% (95% CI: 15.0%–50.1%) within 6–36 months).
Abara *et al.,* 2022 ((8))	Gonorrhea rates in New York City and Philadelphia are among the highest in the United States. Since 2015, ACIP has recommended immunization with a serogroup B meningococcal vaccine for adolescents and young adults aged 16–23 years based on shared clinical decision-making to provide short-term protection against meningococcal disease.	Cohort approach using laboratory-confirmed gonorrhea and chlamydia infections cases among individuals aged 16–23 years identified in sexually transmitted infection surveillance records in New York City and Philadelphia from 2016 to 2018 that were linked to immunization registry records to determine 4CMenB vaccination status at infection. Adjusted VE was estimated using log-binomial regression with generalized estimating equations to account for correlations between multiple infections per patient.	Complete 4CMenB vaccination series VE was 40% (95% CI: 23%–53%) effective and partial vaccination series was 26% effective (95% CI: 12%–37%).
Robinson *et al.,* 2023 ((9))	Mass vaccination campaigns were prompted by serogroup B meningococcal disease outbreaks at University of Oregon in 2015 and Oregon State University in 2016, each used both available meningococcal B vaccines.	Case-control study based on vaccine recipients aged 18–29 years who were reported to Oregon’s ALERT Immunization Information System, linked with gonorrhea cases reported to public health authorities from one month to two years after vaccination.	Overall 4CMenB VE was 47% (95% CI:13%-68%). Among those aged 18–19 years, two-dose VE was 59% (95% CI: 20%–79%).
Bruxvoort *et al.,* 2023 (10)	The Kaiser Permanente Southern-California is a prepaid healthcare system with comprehensive administrative databases including vaccinations and results of laboratory tests.	Cohort study from 2016 to 2020 among individuals 15–30 years of age: recipients of 4CMenB were matched in a ratio of 1:4 to recipients of polysaccharide-conjugate vaccines (MenACWY) and followed for incident gonorrhea using Cox proportional hazards regression, adjusting for potential confounders. The same analysis was conducted with chlamydia as a negative control outcome.	Gonorrhea rates were lower among recipients of 4CMenB vs. MenACWY (VE=46%; 95% CI: 14%–66%), but chlamydia rates were similar between vaccine groups (VE=2%; 95% CI: −17%–18%).
Raccagni *et al.,* 2023 ((11))	In Italy, there is a recommendation for people living with HIV to receive two 4CMenB doses eight weeks apart since 2016.	Unmatched case-control study on men who have sex with men living with HIV, in care at San Raffaele Scientific Institute, Milan, Italy, with gonorrhea, syphilis, chlamydia, or anal human papillomavirus diagnosed between July 2016 and February 2021. For the analysis, cases were people with gonorrhea infection, and controls were people with syphilis, chlamydia, or anal human papillomavirus infection. Logistic regression was used to estimate 4CMenB VE against gonorrhea.	Adjusted VE was 44% (95% CI: 9%–65%).

Abbreviations: ACIP, Advisory Committee on Immunization Practices; HIV, human immunodeficiency virus; VE, vaccine effectiveness; 4CMenB, four-valent serogroup B meningococcal vaccine

### Clinical trials

As shown in **Table 2**, five randomized clinical trials (RCTs) aiming to demonstrate the efficacy of 4CMenB in preventing gonorrhea in different high-risk groups are underway and one has been completed ((12)). In the DOXYVAC trial in France, the incidence of a first episode of gonorrhea (main outcome) was 58.3 per 100 person-years (103 events in 274 participants) in the 4CMenB vaccine group and 77.1 per 100 person-years (122 events in 270 participants) in the no vaccine group (adjusted hazard ratio=0.78 (95% CI: 0.60%–1.01%) ((13)). When the analysis was restricted to participants not receiving doxycycline post-exposure prophylaxis to exclude any interference between the two interventions, the incidence was 76.0 per 100 person-years (40 events in 93 participants) in the 4CMenB vaccine group and 105.3 per 100 person-years (48 events in 90 participants) in the no vaccine group (adjusted hazard ratio=0.76 (95% CI: 0.50%–1.15%). Because this trial was underpowered from the outset and prematurely terminated, a vaccine protection in the 30%–50% range cannot be excluded. Research is also underway to develop a specific *N. gonorrhoeae* vaccine that could induce high-level protection of long duration ((14)). It could, however, take many years to have an *N. gonorrhoeae* vaccine authorized and commercialized in Canada.

**Table 2:** On-going randomized clinical trials aiming to assess the efficacy of the four-valent serogroup B meningococcal vaccine (4CMenB) against gonorrhea infection^a^

Registration	Study title	Participants	Outcome	Sponsor	Source
NTC04415424	*Efficacy Study of 4CMenB (Bexsero®) to Prevent Gonorrhoea Infection in Gay and Bisexual Men (GoGoVax)*	High-risk adults 18–40 years of age (n=730)	*N. gonorrhoeae* infection	Kirby Institute, Australia	https://clinicaltrials.gov/study/NCT04415424
ACTRN12619001478101	*MenGO: Does the licensed meningococcal vaccine Bexsero® provide cross- protection against gonorrhoea in gay and bisexual men?*	High-risk adults ≥18 years of age (n=130)	*N. gonorrhoeae* infection	Gold Coast University Hospital, Australia	https://www.anzctr.org.au/Trial/Registration/TrialReview.aspx?id=376715
NCT04350138	*Safety and Efficacy Study of Meningococcal Group B Vaccine rMenB+OMV NZ (Bexsero) to Prevent Gonococcal Infection*	High-risk adults 18–50 years of age (n=2,200)	*N. gonorrhoeae* infection	National Institute of Allergy and Infectious Diseases (NIAID), United States	https://clinicaltrials.gov/study/NCT04350138
NCT05294588	*Efficacy of Immunization with 4C-MenB in Preventing Experimental Urethral Infection with Neisseria gonorrhoeae*	Healthy males 19–35 years of age (n=140)	Experimental *N. gonorrhoeae* infection	University of North Carolina, Chapel Hill, United States	https://clinicaltrials.gov/study/NCT05294588
NCT05766904	*Efficacy Trial on Meningococcal B Vaccine for Preventing Gonorrhea Infections*	High-risk males 18–50 years of age (n=150)	*N. gonorrhoeae* infection	Chinese University of Hong Kong, China	https://clinicaltrials.gov/study/NCT05766904
NCT04597424	*Combined prevention of sexually transmitted infections (STIs) in men who have sex with men and using oral Tenofovir Disoproxil Fumarate/ Emtricitabine (TDF/FTC) for HIV pre-exposure prophylaxis (PrEP) (DOXYVAC)*	High-risk males ≥18 years of age (n=556)	Sexually transmitted infections, including *N. gonorrhoeae* infection	ANRS, France	https://clinicaltrials.gov/study/NCT04597424

Abbreviations: *N. gonorrhoeae*, *Neisseria gonorrhoeae*; 4CMenB, four-valent serogroup B meningococcal vaccine

^a^ Trials were identified through PubMed, Google Scholar and ClinicalTrial.Gov searches complemented with information provided by GSK

## Discussion

### Biological plausibility

The 4CMenB vaccine that was licensed in Canada in 2014 contains four components: the outer membrane vesicle (OMV) from the NZ98/254 strain, a factor H binding protein (fHbp), neisserial heparin-binding antigen (NHBA), and the *Neisseria* adhesin A (NadA) with the accessory proteins GNA2091 and GNA1030 fused with fHbp and NHBA, respectively, to increase their immunogenicity ((15)). Results of DNA hybridization analyses have shown that *N. meningitidis* and *N. gonorrhoeae* share close to 90% of their genomic identity ((16)). The nucleotide and amino acid sequences of collections of *N. gonorrhoeae* strains were analyzed and compared with antigens included in 4CMenB and their encoding genes. The NHBA-2 peptide in 4CMenB showed moderate sequence identity (73%) to its gonococcal homolog, which is highly conserved within *N. gonorrhoeae* and predicted to be surface expressed (17). The gene encoding NadA is absent in *N. gonorrhoeae* ((18). Although *N. gonorrhoeae* lacks fHbp, it encodes a distinct homolog, Ghfp, which is not expressed on the bacterial surface ((19)). Bioinformatic analyses have found that a homolog of 20 of the 22 major OMV proteins on the 4CMenB vaccine are present in *N. gonorrhoeae* 16 proteins having >90% identity, and 2 proteins having >80% identity ((20)). In mice, 4CMenB was found to elicit antibodies that bind to the surface of *N. gonorrhoeae in vitro* and promote serum bactericidal activity and opsonophagocytic killing activity using human polymorphonuclear leukocytes ((21)). In humans, 4CMenB elicited bactericidal immunoglobulin G (IgG) antibodies to *N. gonorrhoeae* conformational epitopes involving Hep I and Hep II glycosylated lipo-oligosaccharide structures shared between *N. meningitidis* and *N. gonorrhoeae* ((22)).

The evidence drawn from these observational studies meets seven of the causality criteria proposed by Austin Bradford Hill ((23)): i) strength of the association (a large and statistically significant effect size); ii) consistency (reproducibility of results in different studies); iii) specificity of effect (protection against gonorrhea and not against other sexually transmitted infections as shown in a study in Australia) ((24)); iv) temporality (the effect occurs after vaccination); v) biological gradient (effect of one vs two vaccine doses); vi) plausible biological mechanisms and coherence between epidemiological and laboratory findings (discussed in the above section); and vii) analogy (protection was also observed with another OMV meningococcal vaccine in New Zealand) ((25)). Evidence from RCTs is the eighth causality criteria and the most convincing one ((21)). There were many limitations in the only trial which results have been published and we will have to wait for results of other on-going trials to make a final judgement (13).

### Cost-effectiveness evaluations

To investigate the potential public health impact of adolescent 4CMenB vaccination in England, a deterministic transmission-dynamic model of *N. gonorrhoeae* infection among heterosexual 13 to 64 year-olds was developed assuming 31% vaccine efficacy, a six-year span of protection, and 85% uptake, resulting in the prevention of 25% (95% credibility interval: 17%–33%) of heterosexual infections over 70 years (26). No cost-effectiveness evaluation was made in this analysis. In another integrated transmission-dynamic health economic model from England, strategies targeting high-risk groups only were evaluated, including vaccination on attendance for testing in sexual health clinics; vaccination on diagnosis with gonorrhea; or vaccination according to risk offered to patients diagnosed with gonorrhea plus individuals who test negative but report having more than five sexual partners per year ((27)). Results showed that vaccination on attendance would have the fastest and largest impact but at high cost, vaccination on diagnosis would be highly cost-effective but with a much lesser impact, whereas vaccination according to risk would have a similar impact to vaccination on attendance at a fraction of the cost and would likely be cost saving from a health services perspective at the current National Health Services costs (£8 per dose plus £10 for administration). This model was applied to test the targeted vaccination of men who have sex with men in England with one or two 4CMenB doses ((28)). Results indicated that both one- or two-dose strategies would be cost-saving at any uptake level and for a vaccine unit price of £8 per dose plus £10 for its administration.

### Public health implications

In the United Kingdom, the Joint Committee on Vaccination and Immunization (JCVI) has recommended the implementation of a 4CMenB immunization program, the vaccine being offered on an opportunistic basis through specialized sexual health services that have vast experience in assessment and identification of those at increased risk of gonococcal infection ((29)). In the United States, the New York State Department of Health AIDS Institute recommends offering 4CMenB vaccination to patients at high risk of gonorrhea infection (i.e., men who have sex with men and other individuals who have had a bacterial sexually transmitted infection in the prior 12 months, commercial sex workers, and individuals engaging in condomless sex with multiple partners) (30).

## Conclusion

A plausible effectiveness of 4CMenB against *N. gonorrhoeae* infections is not mentioned in the latest version of Canadian product monograph and a new submission by the manufacturer would have to be made to add this indication (15). This hypothesis is briefly mentioned in a recent National Advisory Committee on Immunization statement on meningococcal disease published in the *Canada Communicable Disease Report* (CCDR) in September 2023, and more details on scientific evidence could be easily incorporated into a revision of the Canadian Immunization Guide ((31)). The next step would be a careful evaluation of the integration of 4CMenB into publicly funded provincial/territorial immunization programs for high-risk groups, including the scientific evidence, expected health benefits, budgetary impact, cost-effectiveness, feasibility and acceptability of different vaccination strategies. It is always difficult to extrapolate the country-specific results of economic evaluations and it would thus be interesting to develop a Canadian model or to adapt an existing model to the Canadian context. A first step would be an estimation of the size of high-risk groups and corresponding *N. gonorrhoeae* infections rates in Canada, as well as practical ways to reach these high-risk groups without stigmatization. In the meantime, results of adequately powered randomize RCTs will be available to hopefully support the relevance of a Canadian immunization initiative for the prevention of *N. gonorrhoeae* infections.
